# The effects of an acute bout of ergometer cycling on young adults’ executive function: A systematic review and meta-analysis

**DOI:** 10.1016/j.jesf.2023.07.001

**Published:** 2023-07-17

**Authors:** Tamara S. Dkaidek, David P. Broadbent, Daniel T. Bishop

**Affiliations:** a Centre for Cognitive and Clinical Neuroscience, Brunel University London, Uxbridge, London, UB8 3PH, United Kingdom; b Centre for Sport Research, School of Exercise and Nutrition Sciences, Deakin University, Burwood, Victoria, Australia

**Keywords:** acute exercise, Task switching, Inhibitory control, Working memory

## Abstract

**Purpose:**

The extent to which acute exercise improves executive function (EF) remains indeterminate. The purpose of this systematic review and meta-analysis was to determine the effect of acute ergometer cycling exercise on executive function (EF), including the potential moderating effects of exercise intensity and duration, EF task type, and EF task onset.

**Methods:**

We searched seven electronic research databases using cycling- and cognition-related terms. All 17 studies included were published in the last 10 years and comprised healthy participants aged 18–35 years who completed tasks assessing a variety of EFs before and after cycling exercise lasting 10–60 min. We analyzed 293 effect sizes obtained from 494 individuals (mean age = 22.07 ± 2.46 yrs). Additional analyses were performed, using averaged effect sizes for each separate study to examine the omnibus effect across studies.

**Results:**

There was a positive effect of acute ergometer cycling exercise on response time (RT) in 16 of 17 studies reviewed and a positive effect for response accuracy (RA) in 8 of 14 studies; three studies did not report RA data. Hedges’ g effect sizes [95% CI] for RT ranged from 0.06 [-0.45, 0.56] to 1.50 [0.58, 2.43] and for RA from −1.94 [-2.61, −1.28] to 1.03 [0.88, 1.19].

Bouts of cycling completed at moderate intensities appear to have the greatest effect on RT (Hedges' g = 1.03 [0.88, 1.19]) but no significant effect on RA; bouts with durations of 21–30 min appear to offer the greatest benefits for both RT (Hedges' g = 0.77 [0.41, 1.13]) and RA (Hedges' g = 0.92 [0.31, 1.52]). Effect sizes were greatest for RT in inhibitory control tasks (Hedges' g = 0.91 [0.80, 1.03]) and for RT when EF tasks were completed immediately post-exercise (Hedges’ g = 1.11 [0.88, 1.33]).

**Findings were similar in the omnibus analyses:**

moderate-intensity bouts had the greatest effect on RT, SMD = 0.79 (95% CI [0.49, 1.08]), z = 5.20, p < 0.0001, as did cycling durations of 21–30 min, SMD = 0.87 (95% CI [0.58, 1.15], z = 5.95, p < 0.0001. The greatest benefits were derived for inhibitory control tasks, SMD = 0.70 (95% CI [0.43, 0.98]), z = 5.07, p < 0.04, and when the EF task was completed immediately post-exercise, SMD = 0.96 (95% CI [0.51, 1.41]), z = 4.19, p < 0.001. There were no overall effects on RA.

**Conclusion:**

Our findings indicate that acute bouts of cycling exercise may be a viable means to enhance RTs in immediately subsequent EF task performance, but moderating and interactive effects of several exercise parameters must also be considered.

## 1 Introduction

The effects of acute exercise on executive function (EF) have been extensively researched.^1^, ^2^, ^3^ Executive functions (EFs) have been defined as a set of mental processes that rely on the prefrontal cortex (PFC) region of the brain and have previously been differentiated into three broad components^4^: *cognitive flexibility* or *task switching* (the ability to adapt how we behave based on changes in the environment),^5^ *working memory* (updating old information with novel information)^6^ and *inhibitory control* (the ability to control our cognitions, emotions and behaviours to adapt to our environment).^7^ Our EFs develop through childhood but are still malleable after they peak in early adulthood; the PFC continues to exhibit plasticity in response to both external and internal stimuli throughout the lifespan.^2^ Because changes in healthy adult PFC function can be facilitated by acute exercise,^8^ it is important to identify how we might optimize exercise parameters to maximize this facilitative effect. Several moderators may affect the optimization of exercise interventions, including exercise intensity and duration, the types of EF tasks administered, the post-exercise delay before their completion, and the exercise modality.^9^

According to the American College of Sports Medicine (ACSM), exercise interventions may be categorized as low (37–45% VO_2max_), moderate (46–63% VO_2max_), or high (64–90% VO_2max._) in intensity.^10^ Yerkes and Dodson's^11^ seminal hypothesis proposes an inverted-U relationship between state of arousal and task performance. Researchers have subsequently made predictions based on this hypothesis – namely, that moderate intensity exercise should elicit greater cognitive improvements than low or high intensity. This notion has attracted research attention for half a century,^12^, ^13^, ^14^, ^15^, ^16^ although findings have been inconsistent. Recently, more nuanced mechanistic explanations have emerged, including the role of brain-derived neurotrophic factor (BDNF) in promoting exercise-related neural changes,^2^ and an interoception model that proposes an interaction between the exercisers' perceptions of effort, their motivation to exercise, and their perceptions regarding the availability of personal resources to exercise.^17^ The inconsistent findings regarding the inverted-U hypothesis might be compounded by variation in psychophysiological responses to treadmill versus cycle ergometer exercise protocols^18^; previous research suggests differing effects of these modalities on EF task performance.^19^^,^^20^ For this reason, the current review and meta-analysis is focused exclusively on cycle ergometer protocols.

Previous meta-analyses^1^^,^^3^^,^^19^ suggest that the effects of exercise intensity on cognition may also be contingent on the nature and complexity of EF tasks employed. For example, findings suggest that moderate exercise intensities enhance performance of EF tasks that prioritize speed over accuracy (e.g., Flanker Task), whereas the limited effects on accuracy may be due to the use of EF tasks that are not suitably sensitive to detect performance enhancements.^14^ Importantly, while McMorris and Hale's meta-analysis suggests the enhancements of EF tasks after moderate intensity exercise, they found that heavy exercise resulted in effects close to zero, which may be due to neural noise.^14^ Low complexity tasks can result in ceiling effects for accuracy, and so processing speed is often the variable of interest in such tasks.^21^^,^^24^ Furthermore, the point at which an EF task is administered – i.e., its onset – appears to influence the effect of acute exercise on subsequent EF task performance, although this may depend somewhat on the underlying neurophysiological mechanisms. The catecholamine hypothesis^12^ suggests that catecholamine release occurs constantly throughout an exercise bout, and thus changes may be more influential immediately post-exercise. Conversely, BDNF elevations peak post-exercise, and as such, EF improvements related to circulating BDNF may be sustained for longer. Indeed, the BDNF protein initiates signalling pathways that are implicated in neurogenesis and consequently promotes post-exercise neuroplasticity.^2^^,^^14^ Although the precise effects of BDNF are still being established, it has been suggested that even very brief acute exercise increases circulating peripheral and central BDNF, which has, in turn, been linked to improvements in memory and learning.^2^^,^^14^ However, it has been suggested that moderate and high intensity protocols lead to higher levels of BDNF than low intensity protocols.^27^ Therefore, the effect of EF task onset delays should be considered.

Exercise duration is also purported to moderate the effects of acute exercise on EF. A previous meta-analysis found that exercise durations less than 10 min adversely affected subsequent cognitive performance, whereas longer ones tended to elicit positive effects.^1^ Relatedly, several authors have suggested that exercise bouts lasting approximately 31–40 min yield positive effects,^21^, ^22^, ^23^ although further evidence is needed to determine whether those benefits persist past this duration or, alternatively, whether detriments occur with longer duration.^24^ Most studies to date have examined exercise duration and intensity separately; therefore, we adopted the same approach for this meta-analysis.

The purpose of this systematic review and meta-analysis was to build on the meta-analysis conducted by Chang et al.,^1^ which examined various exercise modalities (e.g., treadmill running, cycling), by analyzing data from literature published in the past decade that examined the effect of a bout of cycle ergometer exercise on EF. We sought to answer two research questions: First, to what extent does a single bout of cycling exercise affect subsequent EF task performance? Second, to what extent do exercise intensity, exercise duration, EF task type, and EF task onset moderate this effect? To address the first question, we focused on within-subject comparisons to reduce variability in the analyses. To facilitate the second, we provide empirically grounded delineations for each category of moderators.

## 2 Methods

### 2.1 Eligibility criteria

The experimental studies included in this review were published in English between January 2012 and December 2022 in Full Text versions, which comprised young adults aged 18–35 years of age with no diagnosed impairments or medical complexities, and included cognitive assessments that assessed working memory, inhibitory control, and/or task switching. Additionally, studies were only included when the authors (a) provided effect sizes for the main effect or provided sufficient information for an effect size to be calculated for separate response time (RT) and/or response accuracy (RA) scores; (b) administered the cognitive assessments pre- and post-exercise; and (c) utilized cycling durations in the range of 10–60 min, at intensities of 37–90% VO_2max_. Elaboration on these criteria can be found in the *Moderators* section below.

### 2.2 Information sources

Searches were conducted in PubMed, Web of Science, Academic Search Complete, CINAHL Plus, APA PsycArticles, APA PsycInfo and SportDiscus databases for dates ranging from January 1st^,^ 2012 to December 7th^,^ 2022. Searches were extracted and reviewed by the researchers. Additional studies were identified by reviewing the References sections of studies retrieved in the search process.

### 2.3 Search strategy

The search terms consisted of the following: (cycl × OR bicycle × OR bike∗) AND (“executive function” OR cogniti∗) AND (planning OR memory OR attention × OR inhibit∗) AND exercise∗.

**Fig. 1:**
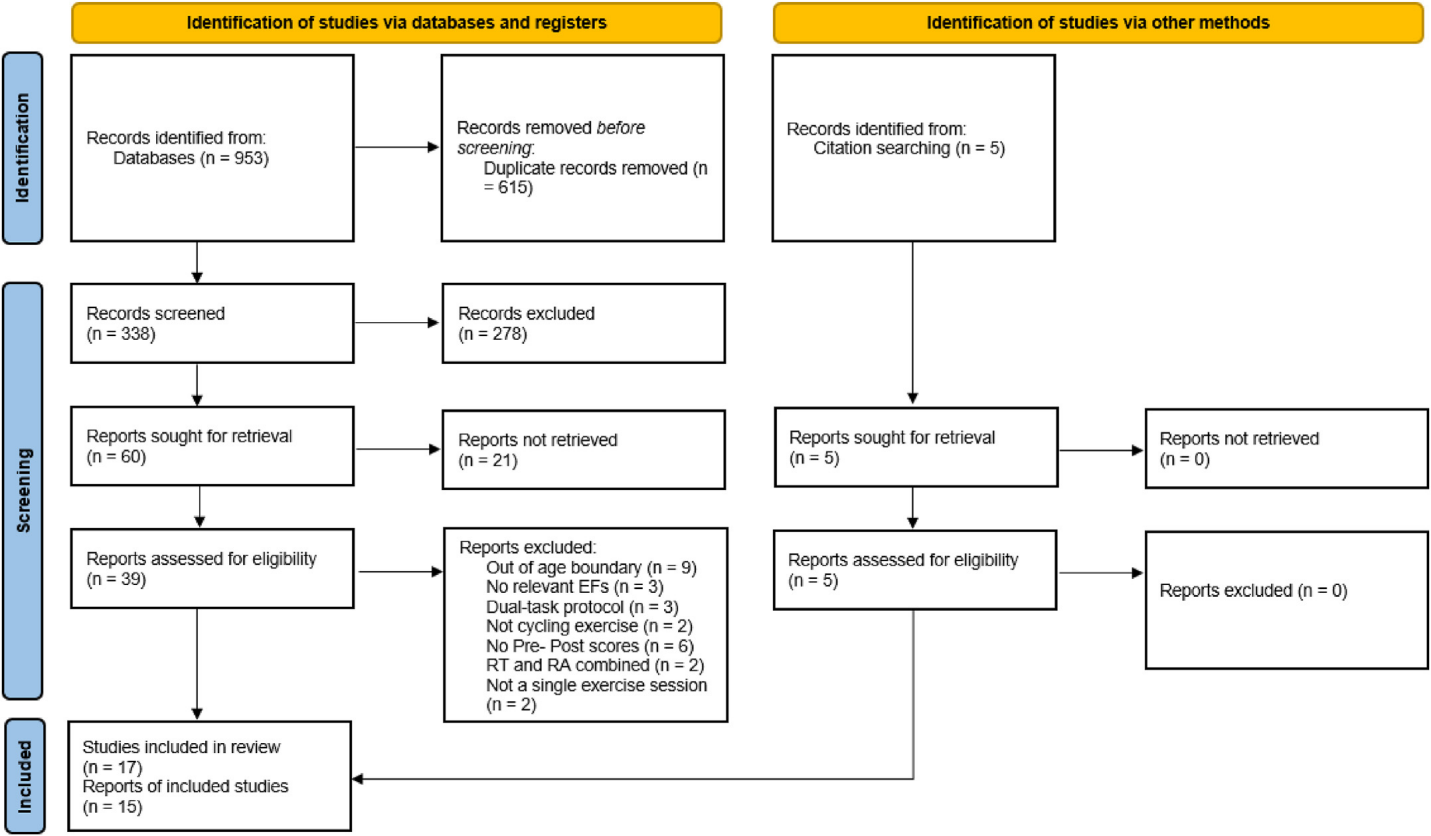
Prisma flow chart

Consistent with previous meta-analyses, the search strategy focused on studies that investigated the effect of an acute bout of cycle ergometer exercise on EFs. Fig. 1 depicts the search strategy we employed. We assessed the eligibility of published articles. First, duplicates were removed. Then, article titles and abstracts were screened based on the eligibility criteria. Records were excluded if the title or abstract indicated that the study included participants outside the age range, if they did not employ an acute single bout of cycling exercise, or if EF task performance was not assessed both before and after an exercise bout. Full-text copies of all retained articles were retrieved and independently assessed for eligibility by all authors before full consensus was reached regarding the articles to be included in the meta-analysis.

### 2.4 Study bias assessment

Risk of bias assessment was conducted using the modified McMaster Critical Review Form.^28^ Table 1 shows the adapted appraisal tool, for which the criteria were reduced from 17 to 12; the excluded criteria apply to all experimental studies, not just randomized control trials. Each met criterion was awarded one point. The criteria were independently rated by the first author and the ratings were discussed with the second and third authors until consensus was reached for each rating.

**Table 1:** Adapted McMaster critical review.

Adapted McMaster Critical Review	
1.	Study purpose
2.	Relevant literature background
3.	Study design stated
4.	Sample described in detail
5.	Sample size justified
6.	Outcome measures are reliable
7.	Outcome measures are valid
8.	Intervention described in detail
9.	Results reported in terms of statistical significance
10.	Appropriate analysis methods
11.	Dropouts reported
12.	Appropriate conclusions based on methods and results

**Table 2:** McMaster critical review form for each study.

Study	1	2	3	4	5	6	7	8	9	10	11	12	N
Aguirre-Loaiza et al. (2019a)^21^	X	X	X	X	X	X	X	X	X	X	X	X	12
Aguirre-Loaiza et al. (2019b)^21^	X	X	X	X	X	X	X	X	X	X	X	X	12
Brown & Bray (2018)^42^	X	X	X	X		X	X	X	X	X		X	10
Chang et al. (2014)^43^	X	X	X	X	X	X	X	X	X	X		X	11
de Diego-Moreno et al. (2022)^44^	X	X	X		X	X	X	X	X	X	X	X	11
Douris et al. (2018)^45^	X	X	X	X		X	X	X	X	X	X	X	11
Hashimoto et al. (2018)^46^	X	X	X	X		X	X	X	X	X		X	10
Miyamoto et al. (2018)^47^	X	X	X	X		X	X	X	X	X		X	10
Oberste et al. (2016)^48^	X	X	X	X	X	X	X	X	X	X		X	11
Sugimoto et al. (2020)^49^	X	X	X	X		X	X	X	X	X		X	10
Tsukamoto et al. (2017a)^24^	X	X	X	X		X	X	X	X	X		X	10
Tsukamoto et al. (2017b)^24^	X	X	X	X		X	X	X	X	X		X	10
Tsukamoto et al. (2016)^22^	X	X	X	X		X	X	X	X	X		X	10
Wang et al. (2015)^50^	X	X	X	X	X	X	X	X	X	X		X	11
Weng et al. (2015)^51^	X	X	X	X		X	X	X	X	X		X	10
Yamazaki et al. (2018)^52^	X	X	X	X		X	X	X	X	X		X	10
Zhu et al. (2021)^23^	X	X	X	X		X	X	X	X	X	X	X	11

**Note:** All criteria are labelled as listed in the Methods section; N = Total Number of points.

The effects of cycling on the moderators (cycling intensity, duration, EF task type and EF task onset) were examined using Studentized residuals and Cook's Distance to detect outliers. Potential outliers were determined based on whether the Studentized Residual value was larger than (100 × [1–0.05/[2 × n]]) the normal distribution, where n is the number of studies. Cook's Distance was considered influential if the value was six times the interquartile range and larger than the median.^29^ Effects were tested using Bonferroni correction with alpha set at 0.05. Publication bias was also assessed via regression and rank correlation tests using the standard error scores of the outcome measures (Table 3). Funnel plots were used to illustrate asymmetry where applicable (Appendix A).^30^

**Table 3:** Publication bias assessments.

**Moderators**		**Fail-Safe N**		**Begg and Mazumdar Rank Correlation**		**Egger's Regression**		**Trim and Fill Number of Studies**	
		Value	P	Value	p	Value	P	Value	p
*RT*		409.00	<.001	0.47	0.01	2.61	0.01	2.00	–
*RA*		0.00	0.374	−0.03	0.91	0.32	0.75	4.00	–
**Moderators**		**Fail-Safe N**		**Begg and Mazumdar Rank Correlation**		**Egger's Regression**		**Trim and Fill Number of Studies**	
		Value	P	Value	p	Value	P	Value	p
*Duration*									
10–20 min	RT	38.00	<.001	−0.05	1.00	0.84	0.40	0.00	–
	RA	22.00	<.001	−0.47	0.27	3.14	0.00	0.00	–
21–30 min	RT	65.00	<.001	−0.20	0.72	1.09	0.28	0.00	–
	RA	33.00	<.001	0.20	0.82	0.12	0.91	0.00	–
31–40 min	RT	88.00	<.001	0.33	0.47	1.24	0.22	1.00	–
	RA	0.00	0.45	−0.80	0.08	1.16	0.25	2.00	–
*Intensity*									
Low	RT	0.00	0.10	0.00	1.00	−0.41	0.68	1.00	–
	RA	–	–	–	–	–	–	–	–
Moderate	RT	161.00	<.001	0.44	0.12	2.11	0.04	2.00	–
	RA	0.00	0.24	0.14	0.77	0.54	0.59	1.00	–
High	RT	79.00	<.001	0.05	1.00	1.07	0.29	0.00	–
	RA	0.00	0.07	−0.33	1.00	−1.97	0.05	0.00	–
*EF Task*									
Working Memory	–	–	–	–	–	–	–	–	–
Inhibitory Control	RT	198.0	<.001	0.30	0.20	1.30	0.20	1.00	–
	RA	25.00	0.001	0.07	0.86	0.92	0.36	0.00	–
Cognitive Flexibility	RT	13.00	<.0w01	−0.33	0.75	−1.05	0.29	2.00	–
	RA	–	–	–	–	–	–		–
*EF Task Onset*									
0–9 min post	RT	247.00	<.001	0.42	0.11	2.45	0.01	1.00	–
	RA	2.00	0.04	−0.14	0.72	−0.48	0.63	3.00	–
10–19 min post	RT	120.00	<.001	0.50	0.11	2.78	0.01	1.00	–
	RA	0.00	0.30	−0.43	0.24	−0.81	0.42	1.00	–
20–29 min post	RT	31.00	<.001	0.33	0.75	0.16	0.88	1.00	–
	RA	14.00	<.001	−0.33	0.75	−0.93	0.36	0.00	–
>30 min post	RT	0.00	0.48	−1.00	0.33	−3.65	<.001	0.00	–
	RA	0.00	0.29	1.00	0.08	0.91	0.36	0.00	–

### 2.5 Synthesis methods

Included studies were those in which intervention group participants’ EF task pre- and post-intervention scores were provided; the latter were acquired either immediately or after a retention period. Descriptive data were collated and inputted into RevMan (v. 5.4.1) software,^31^ which was designed specifically for systematic reviews.

RA and RT were analyzed separately because evidence suggests that low complexity tasks such as the Flanker and Stroop tasks ultimately use processing speed as the criterion performance measure; accuracy measures are included only in these tasks to encourage participant response integrity.^14^ We conducted analyses for overall effects and for each moderator – intensity, duration, EF task type and EF task onset. A positive effect size value corresponded to improvements in EF task performance, whereas a negative value indicated a deterioration in performance.

A random-effects model was applied to the data because the studies included in this review provided estimates of related yet different interventions.^32^ Because of the heterogeneity of methodological approaches and findings, an inverse-variance approach was used to calculate weighted mean effect sizes for each of the studies, which are reported as pre- and post-intervention scores: Hedges’ (adjusted) g effect size. A value of 0.2 is considered a small effect size, 0.5 a medium effect size, and 0.8 a large effect size. Small effect sizes (<0.20) are considered to be trivial regardless of probability level.^33^

Data heterogeneity was characterized in accordance with the Cochrane handbook as chi-squared values reported alongside their associated degrees of freedom (df) and I^2^ values. Chi-squared values indicate whether differences are due to chance.^31^ Notably, a random-effects model is used for this meta-analysis because the studies are different but follow a comparable protocol. Using a random-effects model we can consider that heterogeneity may be based on methodological differences rather than due chance.^32^

#### 2.5.1 Additional analyses - averaged effect sizes

Because some studies’ contributions to the observed effects may be overweighted in the first analysis, an Omnibus Q Analysis was also performed, using the average of effects in each separate study for each moderator. This analysis was also run using a random-effects model, using Q-test and post hoc Z difference tests. The averaged effect sizes for the dependent variables were inputted into Jamovi (2.6.1)^29^ via the MAJOR^34^ plugin. Heterogeneity was estimated using the restricted maximum-likelihood estimator^35^ to yield the Tau^2^ estimate, Q-test^36^ and the I^2^ statistic, as for the previous analysis. These heterogeneity estimates can increase confidence in whether the effect sizes represent true effects in the population or are random.

#### 2.5.2 Moderators

To provide further insight regarding the effect of an acute bout of cycle ergometer exercise on EF, we investigated the effect of four moderating variables on RT and RA (forest plots in Appendix B).

### 2.6 Intensity

Exercise intensities expressed as maximum heart rate (HR_max_) or heart rate reserve (HRR) were converted to percentages of VO_2max_ in accordance with the ACSM: low intensities were defined as those performed at 37–45% VO_2max_, moderate intensities at 46–63% VO_2max_, and high intensities at 64–90% VO_2max._

### 2.7 Duration

Exercise durations were classified as follows: 10–20 min, 21–30 min, 31–40 min, and greater than 40 min. This categorization is comparable to those identified by Chang et al.^1^ in their meta-analysis, which showed that durations of 0–10 min elicited small negative effects, 11–20 min brought about small positive effects, and greater than 20 min yielded large positive effects. However, we selected a minimum duration of 10 min in accordance with the ACSM stipulation that exercise program should last for 10–60 min, notwithstanding moderating effects of exercise intensity.^24^ We anticipated that this categorization would afford greater differentiation of exercise intensities and would consequently enable us to better understand whether an inverted-U relationship exists.

### 2.8 EF task type

Working memory tasks comprised the Trail Making Test^37^ (TMT) and the n-Back task.^38^ Inhibitory Control Tasks comprised the Stroop Color and Word Test^39^ (Stroop) and the Eriksen Flanker Task^40^ (Flanker). The Wisconsin Card Sorting Task^41^ (WCST) was the only task switching measure used.

### 2.9 EF task onset

The EF task onset was defined as the period of delay, in minutes, between cessation of the exercise bout and commencement of the EF task. The delay periods were classified as follows: 0–9 min (*immediate*), 10–19 min (*short delay*), 20–29 min (*moderate delay*), and greater than 30 min (*long delay*). We based this categorization on those used in published studies, although descriptions differ slightly (e.g., Post 0, Post 10, Post 20, Post 30).^22^^,^^24^^,^^48^

## 3 Results

### 3.1 Study selection

An initial search of the databases identified 953 nonduplicate records (Fig. 1). After reviewing the titles and abstracts, 44 full-text reports were screened based on the eligibility criteria. Of these, 15 papers comprising 17 empirical studies met the inclusion criteria.

### 3.2 Study characteristics

The 17 included studies comprised 494 participants, of whom 59 were women and 254 were men; sex was not stated for some samples, and so could not be determined for 181 participants. The participants’ average age was 22.07 ± 2.46 years. Two-hundred and ninety-three effect sizes were analyzed.

### 3.3 Metabias assessment

**Fig. 2:**
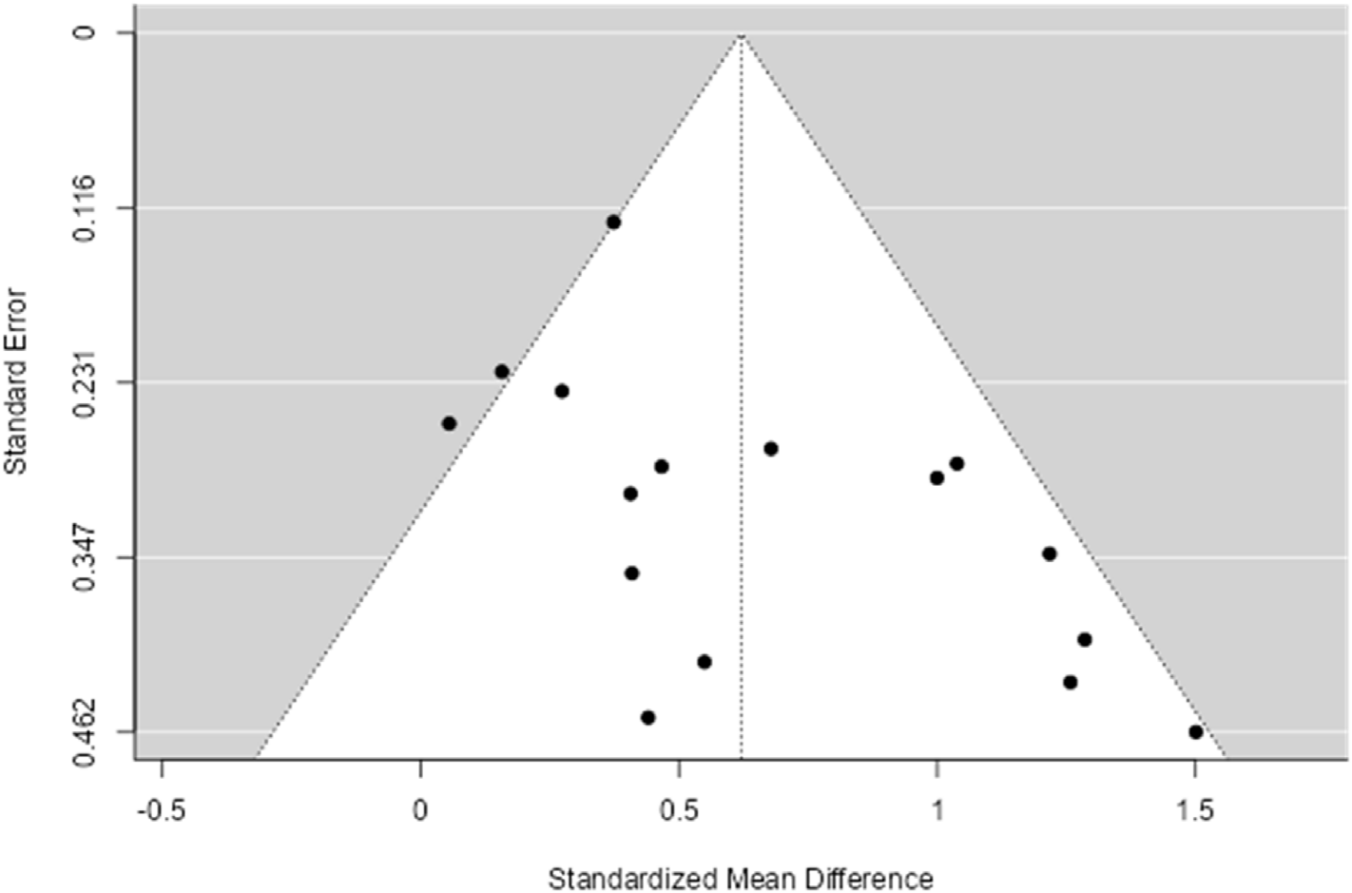
Funnel plot – response time (overall).

**Fig. 3:**
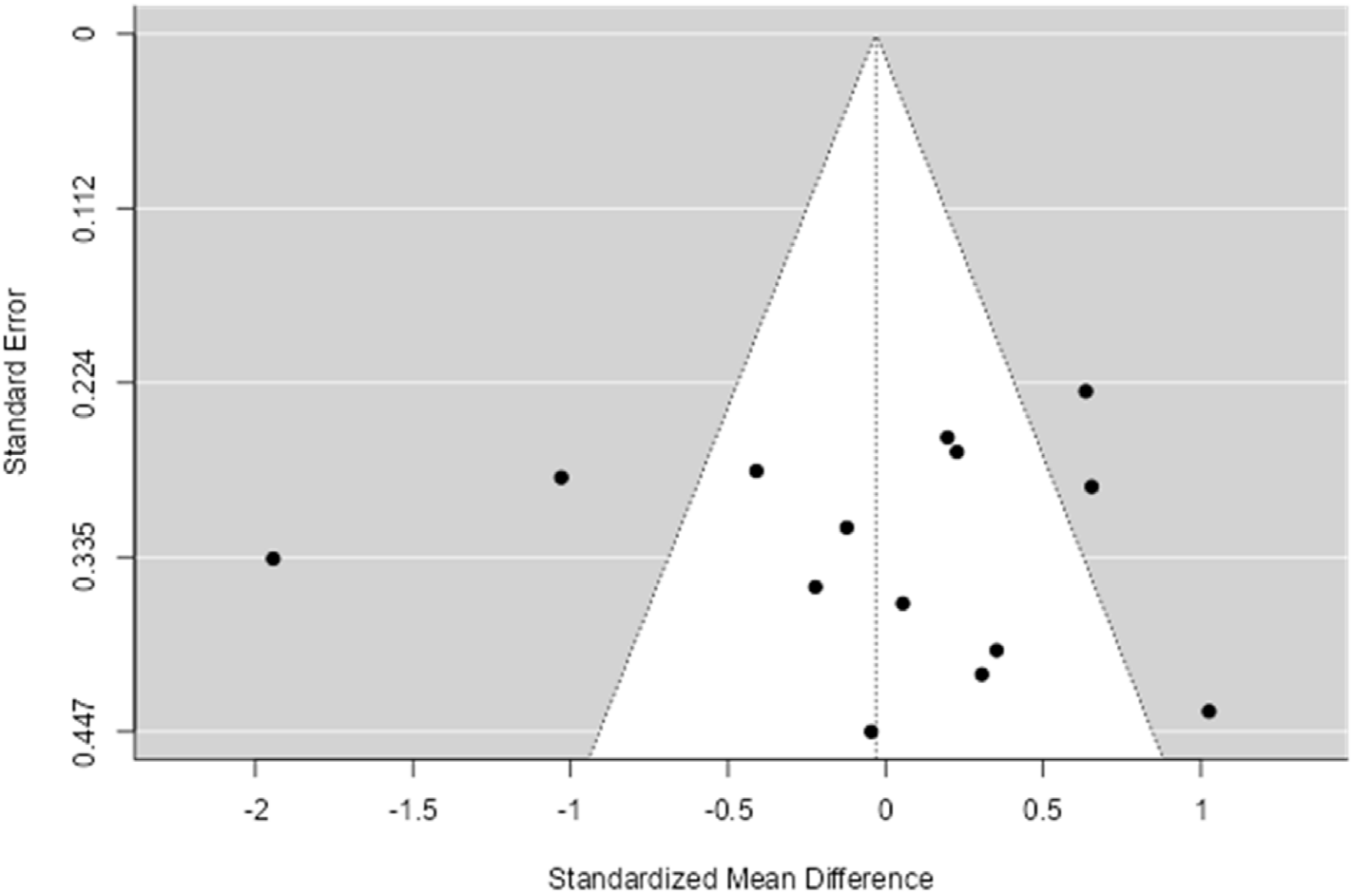
Funnel plot - response accuracy (overall)Table 3. Table.

Table 2 represents the modified McMaster Critical Review rankings of the studies, all of which scored 9–12 out of 12. Publication bias was also assessed according to asymmetry in the associated funnel plots for the overall effects on EF response time and accuracy, and the effects on those measures for each moderator (Fig. 2, Fig. 3, Appendix B). Finally, Publication Bias assessments were run using multiple tests: Fail-safe N, Begg and Mazumdar Rank Correlation, Egger's Regression, and the Trim and Fill Number of Studies (Table 3).

### 3.4 Overall RT

A total of n = 16 studies were included in this analysis**.** There was a moderate overall effect of an acute bout of cycling exercise on RT, Hedges’ g = 0.61 (95% CI [0.41, 0.82]), df = 16 (P = 0.01), I^2^ = 49%. The I^2^ value of 49% indicates moderate heterogeneity: approximately half of the variability in the observed effect sizes is based on between-study differences.^3^

#### 3.4.1 Additional analyses - averaged effect sizes

**Fig. 4:**
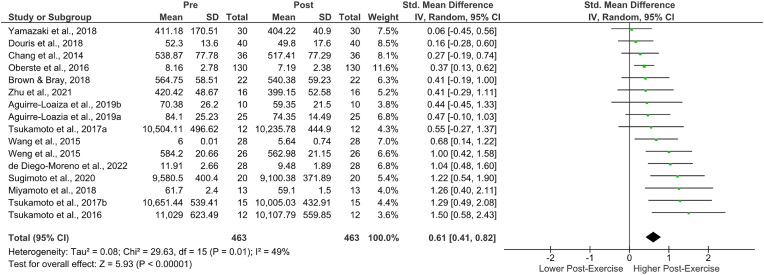
RTs – forest plot.

The SMD ranged from 0.06 to 1.50, with the majority of estimates resulting in a positive effect (z = 5.93, p = < 0.0001). The Q-test determined that the true outcome was heterogeneous; however, both the true outcomes and the estimated outcome of each study were positive (Q 15 = 30.12, p = 0.01, Tau^2^ = 0.08; please refer to Fig. 4 for the forest plot, Table 4, Table 5 for random-effects model statistics and Table 6 for heterogeneity statistics). Based on the Studentized Residuals, there are no outliers in the context of this model, and both the regression and rank correlation tests showed potential funnel plot asymmetry (Fig. 2 and Table 3)

**Fig. 5:**
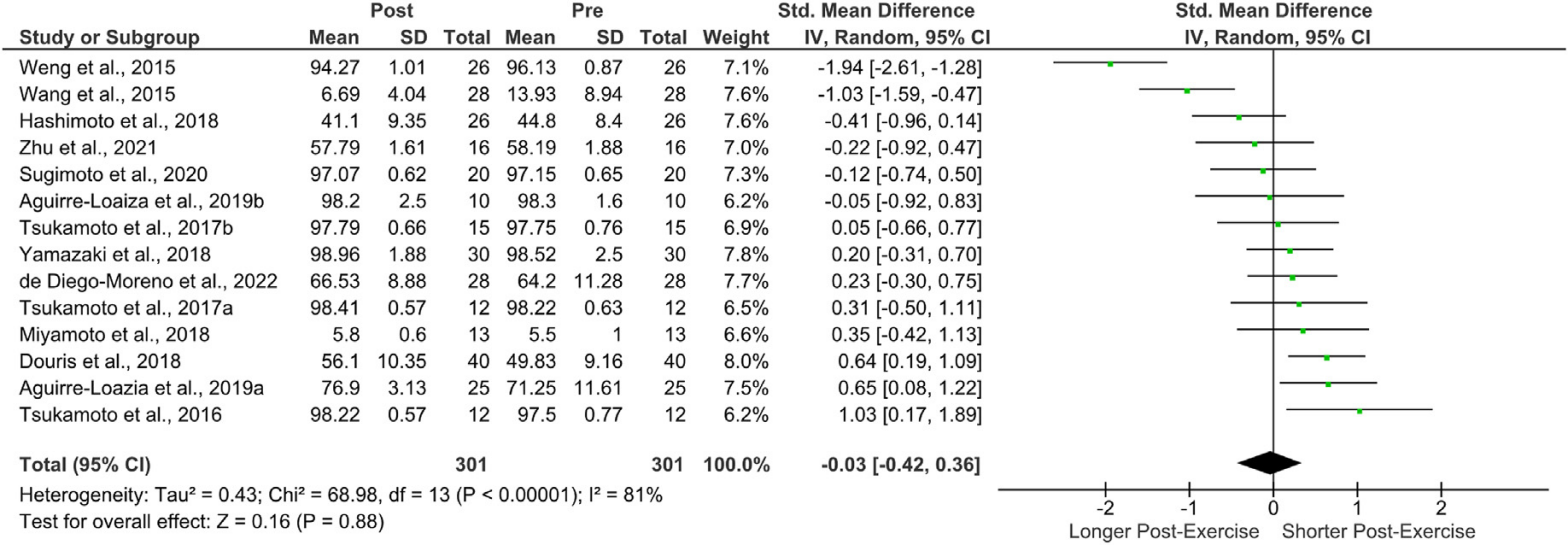
RAs – forest plot.

**Table 4:** Effects, by moderator – individual effect sizes.

Paradigm	N		Analysis			
			Chi-squared	df	Hedge's g	95% CI
*Intensity*						
Low	628	RT	103.43	37	0.35	0.16, 0.55
	408	RA	118.41	27	−0.48	−0.80, −0.15
Moderate	1464	RT	327.83	86	1.03	0.88, 1.19
	1332	RA	369.43	81	0.03	−0.14, 0.20
High	874	RT	153.28	46	0.97	0.78, 1.16
	416	RA	222.53	25	−0.68	−1.13, −0.22
*Duration*						
10–20 min	1550	RT	348.20	95	0.79	0.65, 0.94
	1249	RA	551.20	77	−0.28	−0.51, −0.06
21–30 min	254	RT	39.44	11	0.77	0.41, 1.13
	275	RA	114.71	11	0.92	0.31, 1.52
31–40 min	1189	RT	257.92	64	0.99	0.81, 1.17
	740	RA	257.73	49	−0.09	−0.34, 0.16
>40 min	100	RT	13.58	3	0.40	−0.21, 1.00
	50	RA	0.43	1	−0.66	−1.06, −0.25
*EF Task Type*						
Working Memory	240	RT	0.52	7	0.07	−0.11, 0.25
	324	RA	8.04	10	−0.10	−0.26, 0.05
Inhibitory Control	2539	RT	553.10	156	0.91	0.80, 1.03
	1993	RA	936.04	132	−0.1	−0.34, 0.01
Task-switching	260	RT	8.85	10	0.71	0.53, 0.88
	41	RA	4.57	1	0.20	−0.80, 1.21
*EF Task Onset*						
Immediately post-exercise	968	RT	264.21	53	1.11	0.88, 1.33
	817	RA	309.62	43	0.11	−0.17, 0.39
Short delay	1124	RT	181.92	54	0.85	0.69, 1.02
	560	RA	215.41	35	−0.12	−0.43, 0.19
Moderate delay	435	RT	63.87	29	0.87	0.66, 1.08
	435	RA	151.40	29	−0.52	−0.85, −0.20
Long delay	362	RT	116.67	23	0.40	0.05, 0.76
	435	RA	348.62	29	−0.09	−0.62, 0.44

**Table 5:** Averaged effect sizes.

Overall		SMD	SE	Z	P	CI Lower	CI Upper
*RT*		0.61	0.11	5.84	<.001	0.40	0.82
*RA*		−0.03	0.20	−0.16	0.88	−0.42	0.36
**Moderator**		**Estimate**	**SE**	**Z**	**p**	**CI Lower**	**CI Upper**
*Intensity*							
Low	RT	0.19	0.25	0.77	0.44	−0.29	0.67
	RA	–	–	–	–	–	–
Moderate	RT	0.79	0.51	5.20	<.001	0.49	1.09
	RA	0.08	0.12	0.63	0.53	−0.16	0.32
High	RT	0.72	0.14	5.24	<.001	0.45	0.99
	RA	−0.39	0.56	−0.69	0.49	−1.49	0.72
*Duration*							
10–20 min	RT	0.59	0.25	2.34	0.02	0.10	1.09
	RA	−0.58	−1.69	−1.60	0.11	−1.29	0.13
21–30 min	RT	0.87	0.15	5.95	<.001	0.58	1.15
	RA	0.77	0.45	1.72	0.09	−0.11	1.65
31–40 min	RT	0.21	0.21	4.58	<.001	0.56	1.39
	RA	−0.00	0.16	−0.02	0.98	−0.32	0.32
*EF Task Type*							
Working Memory	–	–	–	–	–	–	–
Inhibitory Control	RT	0.70	0.14	5.02	<.001	0.43	0.98
	RA	0.39	0.32	1.21	0.23	−0.24	1.03
Task-switching	RT	0.62	0.17	3.71	<.001	0.29	0.95
	RA	–	–	–	–	–	–
*EF Task Onset*							
0–9 min post	RT	0.96	0.23	4.19	<.001	0.51	1.41
	RA	0.20	0.33	0.62	0.54	−0.44	0.84
10–19 min post	RT	0.80	0.19	4.25	<.001	0.43	1.17
	RA	−0.07	0.18	−0.36	0.72	−0.43	0.29
20–29 min post	RT	0.95	0.20	4.84	<.001	0.56	1.33
	RA	−0.64	0.19	−3.35	<.001	−1.01	−0.27
>30 min post	RT	−0.33	1.21	−0.28	0.78	−2.71	2.04
	RA	−0.11	0.19	−0.61	0.54	−0.48	0.25

**Table 6:** Heterogeneity statistics.

Overall		Tau	Tau^2^	I^2^	H^2^	Df	Q	p
*RT*		0.29	0.08 (SE = 0.06)	49.00%	2.10	15.00	30.12	0.01
*RA*		0.67	0.43 (SE = 0.22)	81.00%	5.48	13.00	70.11	<.001
**Modality**		**Tau**	**Tau****^2^**	**I****^2^**	**H****^2^**	**df**	**Q**	**p**
*Intensity*								
Low	RT	0.38	0.14 (SE = 0.20)	59.71%	2.48	3.00	7.49	0.06
	RA	–	–	–	–	–	–	–
Moderate	RT	0.30	0.09 (SE = 0.10)	46%	1.85	8.00	14.81	0.06
	RA	0.00	0 (SE = 0.056)	0%	1.00	6.00	3.24	0.78
High	RT	0.13	0.02 (SE = 0.07)	13.57%	1.16	6.00	8.04	0.24
	RA	0.90	0.80 (SE = 0.95)	85.17%	6.74	2.00	11.16	0.00
*Duration*								
10–20 min	RT	0.56	0.31 (SE = 0.26)	69.89%	3.32	6.00	19.58	0.00
	RA	0.80	0.64 (SE = 0.49)	82.81%	5.82	5.00	21.77	<.001
21–30 min	RT	0.00	0 (SE = 0.08)	0%	1.00	5.00	5.05	0.41
	RA	0.92	0.85 (SE = 0.71)	85.72%	7.01	4.00	29.83	<.001
31–40 min	RT	0.36	0.13 (SE = 0.17)	49.19%	1.968	5.00	9.80	0.08
	RA	0.00	0 (SE = 0.10)	0%	1.00	4.00	1.92	0.75
*EF Task Type*								
Working Memory	–	–	–	–	–	–	–	–
Inhibitory Control	RT	0.33	0.11 (SE = 0.10)	45.85%	1.85	11.00	20.47	0.04
	RA	0.95	0.901 (SE = 0.492)	87.4%	7.93	9.00	64.17	<.001
Task-switching	RT	0.00	0 (SE = 0.09)	0%	1.00	3.00	1.58	0.66
	RA	–	–	–	–	–	–	–
*EF Task Onset*								
0–9 min post	RT	0.63	0.40 (SE = 0.25)	76.93%	4.34	9.00	36.58	<.001
	RA	0.86	0.731 (SE = 0.46)	86.79%	7.57	7.00	46.05	<.001
10–19 min post	RT	0.40	0.16 (SE = 0.15)	58.70%	2.42	7.00	16.96	0.02
	RA	0.28	0.08 (SE = 0.13)	38.27%	1.62	5.00	8.30	0.14
20–29 min post	RT	0.00	0 (SE = 0.13)	0%	1.00	3.00	3.30	0.35
	RA	0.00	0 (SE = 0.12)	0%	1.00	3.00	3.46	0.33
>30 min post	RT	2.05	4.20 (SE = 4.40)	96.07%	25.47	2.00	34.26	<.001
	RA	0.00	0 (SE = 0.11)	0%	1.00	3.00	1.46	0.69

#### 3.5 Overall RA

A total of n = 14 studies was included in these analyses. When assessing all effect sizes, there was no effect of an acute bout of cycling exercise on RA, Hedges' g = −0.03 (95% CI [−0.42, 0.36)]), df = 14 (P < 0.00001), I^2^ = 81%, z = 0.16, p = 0.88 with no significant difference from zero. The Q-test determined that the true outcome was heterogeneous; however, the average outcome is negative (Q (13) = 70.10, p = <0.0001), Tau^2^ = 0.43; please refer toFig. 5 for the forest plot, Table 4, Table 5 for random-effects model statistics and Table 6 for heterogeneity statistics). Based on the Studentized Residuals and Cook's Distance, the Weng et al. (2015)^51^ study may be a potential outlier in this model and may be too influential. Both the regression and rank correlation tests showed no funnel plot asymmetry (Table 3 and Fig. 3).

### 3.6 Moderators

Table 4 shows the effect sizes for RA and RT, by moderator. Forest Plots for each individual moderator for both RT and RA can be found in Appendix B.

### 3.7 Intensity

Moderate intensity exercise resulted in the greatest improvements in RT, Hedges' g = 1.03 (95% CI [0.88, 1.19]). Similar large effects were found for RT in high intensity exercise, Hedges' g = 0.97 (95% CI [0.78, 1.16]), whereas low intensity exercise yielded smaller improvements, Hedges' g = 0.35 (95% CI [0.16**,** 0.55]). High intensity exercise yielded a negative effect on RA, Hedges' g = −0.68 (95% CI [−1.13, −0.22]) and low intensity exercise elicited a negative effect on RA, Hedges’ g = −0.48 (95% CI [−0.80, −0.15]) Moderate intensity cycling had no significant effects on RA.

#### 3.7.1 Additional analyses - averaged effect sizes

Moderate intensity (n = 9 studies) showed the most significant positive effect on EF task RT, SMD = 0.79 (95% CI [0.49, 1.08], z = 5.20, p < 0.0001), however, the Q-test was not significant, which indicates that there may be heterogeneity. Following, High intensity cycling (n = 7 studies) also showed a positive effect on EF task RT, SMD = 0.72 (95% CI [0.45, 0.99]), z = 5.24, p < 0.0001. Low intensity (n = 4) cycling showed no significant effects on RT, and none of the intensities had a significant post-exercise effect on EF task RA.

### 3.8 Duration

The greatest improvements in RT were found for 31–40 min of cycling, Hedge's g = 0.99 (95% CI [0.81, 1.17]). Less improvement was found for 10–20 min of cycling, Hedge's g = 0.79 (95% CI [0.65, 0.94]), and 21–30 min, Hedges' g = 0.77 (95% CI [0.41, 1.13]). Bouts of 40 min or longer yielded no significant effect on EFs. The greatest improvements in RA were after 21–30 min of cycling, Hedges' g = 0.92 (95% CI [0.31, 1.52]). All other durations yielded a negative effect on RA; the smallest decline in performance occurred after 10–20 min of cycling, Hedge's g = −0.28 (95% CI [−0.51, −0.06]), and the greatest decline occurred after 40 min or longer, Hedge's g = −0.66 (95% CI [−1.06, −0.25]. There was no effect of cycling bouts lasting 31–40 min on EF performance.

#### 3.8.1 Additional analyses - averaged effect sizes

After averaging dependent effect sizes by individual study, 21–30 min yielded a positive effect on post-exercise EF RT (n = 6 studies), SMD = 0.87 (95% CI [0.58, 1.15]), z = 5.95, p < 0.0001, 21–30 min of cycling (n = 5 studies) yielded a positive but non-significant effect. A duration of 31–40 min of cycling yielded a positive effect on EF RT, SMD = 0.21 (95% CI [0.56, 1.39], z = 4.58, p < 0.0001. However, the effect of 31–40 min of cycling on post-exercise EF RA (n = 5 studies) was negative and non-significant. 11–20 min of cycling (n = 7 studies) yielded a positive effect on EF RT, SMD = 0.59 (95% CI [0.10, 1.09], z = 2.34, p = 0.02, and yielded a negative effect on RA.

### 3.9 EF task type

An acute bout of ergometer cycling exercise elicited the greatest improvement in RT for inhibitory control, Hedges' g = 0.91 (95% CI [0.80, 1.03]), followed by task-switching, Hedges’ g = 0.71 (95% CI [0.53, 0.88]). There was no significant effect on working memory tasks performance. There was no significant effect of cycling on RA for any EF tasks.

#### 3.9.1 Additional analyses - averaged effect sizes

When averaging dependent effect sizes by individual study, acute cycling yielded the most beneficial effects on inhibitory control RT (n = 12 studies), SMD = 0.70 (95% CI [0.43, 0.98]), z = 5.07, p < 0.04. There was an insignificant effect of acute cycling on RT in tasks assessing inhibitory control (n = 10 studies). Acute cycling did not have a significant effect on RT in tasks assessing task-switching (n = 4 studies). There was a positive, non-significant effect of acute cycling exercise on RA for inhibitory control tasks. There was insufficient data to run an analysis for RA for task-switching (n = 2) and working memory tasks (n = 2).

### 3.10 EF task onset

Completion of EF tasks immediately post-exercise resulted in the greatest improvements on RT, Hedges' g = 1.11 (95% CI [0.88, 1.33]). Lesser improvements in RT were found after a short delay, Hedge's g = 0.85 (95% CI [0.69, 1.02]), and after a moderate delay, Hedge's g = 0.87 (95% CI [0.66, 1.08]), but the least improvement in RT was observed after a long post-exercise delay, Hedge's g = 0.40 (95% CI [0.05, 0.76]). There was a decline in EF task performance after a moderate delay, Hedge's g = −0.52 (95% CI [−0.85, −0.20]) and no significant effects on EF in any other delay category.

#### 3.10.1 Additional analyses - averaged effect sizes

When averaging dependent effect sizes by individual study, the greatest improvements in RT were found immediately post-exercise (n = 4 studies), estimated SMD = 0.96 (95% CI [0.51, 1.41]), z = 4.19, p < 0.001. Similar improvements in RT were found after a moderate post-exercise delay, SMD = 0.95 (95% CI [0.56, 1.33]), z = 4.84, p < 0.0001, and after a short post-exercise delay, SMD = 0.80 (95% CI [0.43, 1.17], z = 4.25, p > 0.001. However, a long delay yielded no effect on RT. There were no significant effects of an acute bout of cycling on RA, regardless of administration time.

## 4 Discussion

This meta-analysis aimed to (i) determine the effect of an acute bout of ergometer cycling exercise on EF and (ii) obtain some insight regarding the influence of established moderators on this effect. The findings of this current review support the inverted-U hypothesis: moderate intensity exercise protocols elicited the greatest EF task performance benefits for RT. For RT, there was a marginally smaller effect size after high intensity exercise and a minimal effect after low intensity exercise. An acute bout of cycling exercise had no effect on RA, irrespective of intensity. A second analysis was conducted to consider the effect of sample size and study weight on the outcomes, which averaged the effect sizes of each separate study.^1^ The analysis of moderators suggests that optimal exercise intensities may be 46–63% of VO2max, optimal durations are approximately 21–30 min, and optimal EF improvements are manifested immediately post-exercise.

These findings may be contingent on the type of EF task employed. The EF task with the greatest RT improvements post-exercise was inhibitory control, with benefits also evident for task-switching RT tasks; however, there were no significant effects of acute cycling on RA across any EF task type. This is somewhat consistent with Yerkes and Dodson's claim that lower arousal levels are required for complex tasks. Still, high arousal levels may be preferable for simple ones.^11^ McMorris and Hale noted that, when performing tasks such as the Flanker Task, the individual must choose their response while preparing to move and select their answer. If the individual decides to focus on increasing their speed, this may be at the cost of accuracy, and RT seems to be favoured over RA.^14^ Another reason for the effects of RA and RT is that, according to the catecholamine hypothesis, acute exercise-induced increases in catecholamines could positively affect RT but may cause neural noise that results in performance decrements.^12^ For example, increased catecholamine levels have been shown to affect RT positively. However, the resultant noise in the dorsolateral prefrontal cortex (DLPFC) may impair task accuracy by reducing its capacity to prevent interference (e.g., from immediately preceding items in a 2-back task).^53^ Although the previous study has only assessed the relationship between acute exercise and decline in RA during working memory tasks, the researchers suggest that this finding could be extended to other cognitive tasks as well, which leads to the importance of assessing RT and RA separately in future studies.^14^^,^^53^

A negative or more negligible effect on RA compared to RT in healthy young adults could be explained in the context of McMorris's interoception theory.^54^ This theory suggests that perceptions of fatigue associated with high-intensity exercise may offset the physiological benefits of EF. The individual may perceive the task as having a high effort cost, resulting in decreased activation of dopaminergic projections from the nucleus accumbens to the dorsolateral prefrontal cortex, which culminates in lower motivational salience for the task, i.e., reduced incentive to be accurate.^55^ Moreover, short task durations limit the possibility of in-task learning, so the individual's perceptions of the effort required in the cognitive task may be higher in the exercise condition. Future research in this area should consider affective responses, such as perceived exertion, alongside objective measures, such as VO2max, when determining the influence of exercise intensity on executive function task performance.

Exercise duration seems to influence the extent of improvement in EF task performance. The current analysis shows that 21–30 min of exercise elicited improvements in EF task performance when considering RT. There were no significant effects of cycling exercise on RA at any duration. The timeframe of 21–30 may be optimal for triggering physiological mechanisms that promote neuroplasticity and cognitive optimization, but it could be contingent on exercise intensity; the combined and interacting contributions of exercise intensity and duration – recently described as exercise volume^22^ – may be a more accurate way of specifying target thresholds for neuroplastic changes and EF enhancement.^23^, ^24^, ^25^, ^26^ For example, in their volume-controlled analyses, Tsukamoto and colleagues^22^ found that exercise-induced benefits were sustained for longer retention periods after moderate intensity exercise than low intensity, volume-matched exercise, and longer-duration moderate intensity exercise seemed to prolong EF improvements. This finding indicates that sustained arousal may be influential in determining prolonged acute exercise-induced EF improvements. However, previous research has tended not to examine the interactive effects of two or more moderators, although there is some evidence of positive effects for short bouts at very high intensities.^44^^,^^56^

Exercise yielded a positive effect on RT in all task types. However, the most considerable effect was seen for inhibitory control and task-switching measures; there was no effect for working memory tasks. For RA, there were no effects for any EF task types. These findings agree with McMorris and Hale's findings, who noted that inhibition and working memory tasks might not be complex enough to assess RA.^14^ As reflected in many of the studies in this review, working memory or inhibitory control is typically examined in isolation. One exception is the study by Weng and colleagues,^51^ who found significant enhancement in working memory after 30 min of moderate intensity exercise, using the 2-Back condition of the facial n-Back but no effect for inhibitory control as measured using the Flanker Task. It would be prudent for future studies to directly compare performance on two different EF task types using equivalent experimental designs and samples.

The findings in this review suggests that the optimal EF task onset ranges from immediately to 9 min post-exercise.^2^ However, this finding is based on five studies that comprise varying ratios of exercise intensities with different durations.^22^^,^^24^^,^^43^^,^^47^^,^^53^ To account for such variability, it is important to employ volume-controlled protocols as done by Tsukamoto and colleagues.^22^ The optimal improvement in EF task performance that occurred immediately post-exercise suggests that physiological changes that influence RT on EF tasks (i.e., peripheral and central BDNF, heart rate and catecholamine concentrations) may subside quickly after the exercise session.^44^

### 4.1 Implications for cycling as an intervention

This review and meta-analysis suggest that an acute bout of cycling exercise may improve young adults' subsequent performance of EF tasks – specifically those dependent on working memory, shifting, and inhibitory control. These EFs serve an essential purpose in our everyday lives, enabling us to pay attention, regulate emotions, make decisions and retain information.^57^ Accordingly, the relationship between EF task performance and academic achievement is established^58^^,^^59^ cycling could be promoted as a mode of active school travel, and brief cycling exercise sessions could also be incorporated into school timetables to maximize students’ academic performance in class. However, additional research is required in this regard.^59^ Ergometer protocols may also be helpful for examining the potential effects of physical and cognitive exercise on EF task performance. For example, the greater stability of ergometer cycling relative to treadmill running may facilitate safe performance of a concurrent secondary task.

### 4.2 Limitations

This review has a few limitations to consider. First, our sample was restricted to healthy young adults and is therefore not generalisable to individuals outside this cohort. However, this approach mitigated the potential confounding effect of participant age, in line with previous recommendations.^60^ Second, studies were only included if cycling ergometer exercise was the sole intervention; all those that comprised one or more other intervention components (e.g., caffeine consumption) were excluded. Consequently, the applicability of the findings in this review may not extend to real-life cycling, which occurs under various circumstances, such as those in which caffeine has been imbibed prior to a cycle journey (e.g., the morning commute).

In this meta-analysis, we acknowledged the potential mediating effect of individual differences, such as age and health status, on EF performance in our inclusion criteria. However, we did not account for participants' sex, fitness levels, their perceived exertion during exercise or other individual differences because insufficient information was provided in previous research to characterise samples in these respects effectively. For example, according to McMorris' model, motivational factors may affect an individual's perception of effort/the perceived costs of exercising.^15^

## 5 Conclusion

This meta-analysis, which included 293 effect sizes across 17 studies, found that when considering both RT and RA, the greatest improvements in EF task performance result from acute cycling bouts at moderate intensities for durations ranging from 21 to 30 min. EF task performance was greatest immediately post-exercise. The EF component that exhibited the greatest post-exercise improvements was inhibitory control. These findings lend support for the use of cycling-based interventions to enhance subsequent cognitive performance.

## Authorship

Category 1.

Conception and design of study: Dr David Broadbent, Dr Daniel Bishop, Tamara Dkaidek. Acquisition of data: Tamara Dkaidek. Analysis and/or interpretation of data: Dr David Broadbent, Dr Daniel Bishop, Tamara Dkaidek.

Category 2.

Drafting the manuscript: Tamara Dkaidek. revising the manuscript critically for important intellectual content: Dr David Broadbent, Dr Daniel Bishop.

Category 3.

Approval of the version of the manuscript to be published (the names of all authors must be listed):

## Declaration of competing interest

A conflict of interest occurs when an individual's objectivity is potentially compromised by a desire for financial gain, prominence, professional advancement or a successful outcome. *JESF* Editors strive to ensure that what is published in the Journal is as balanced, objective and evidence-based as possible. Since it can be difficult to distinguish between an actual conflict of interest and a perceived conflict of interest, the Journal requires authors to disclose all and any potential conflicts of interest.

## Figures and Tables

**Figure:**
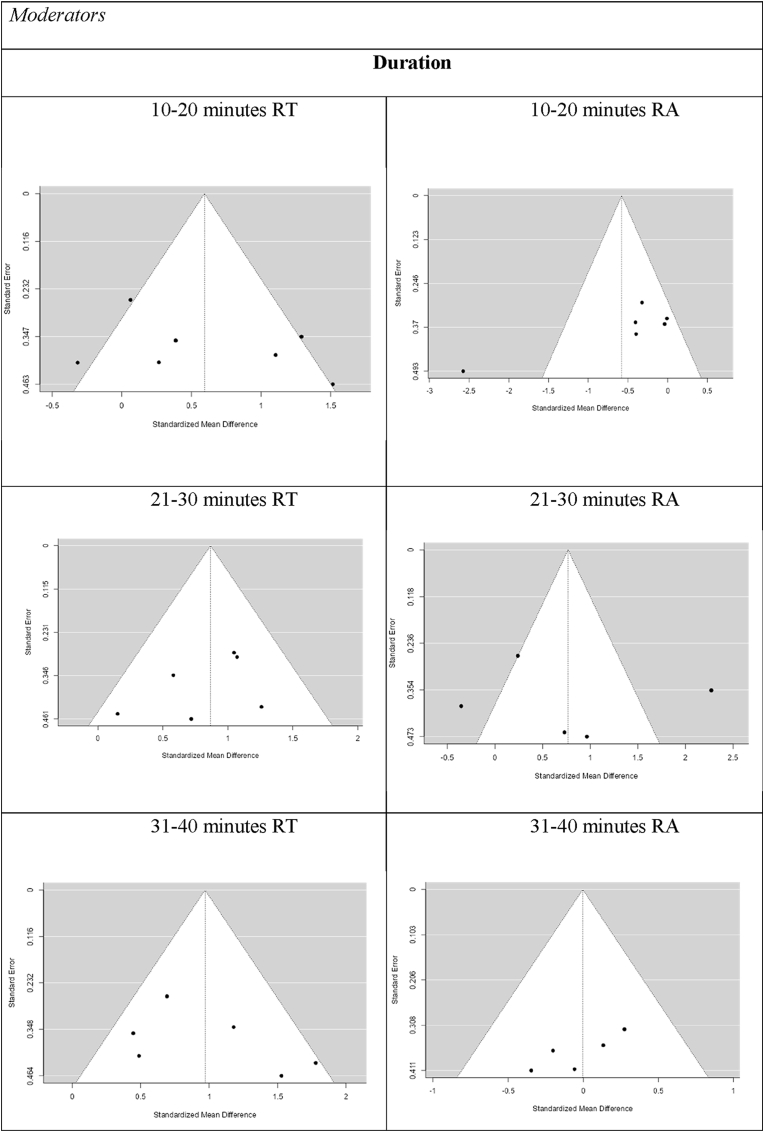

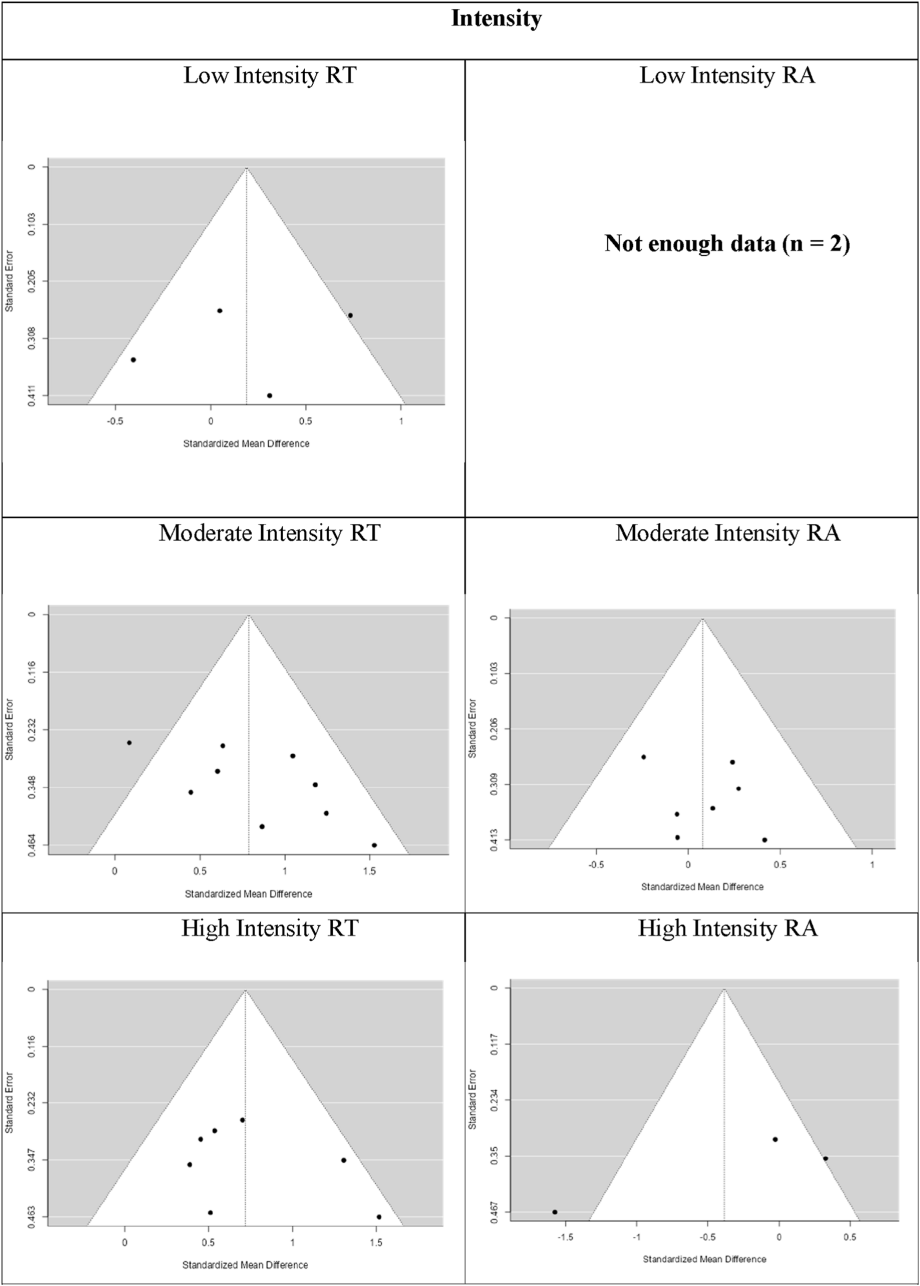

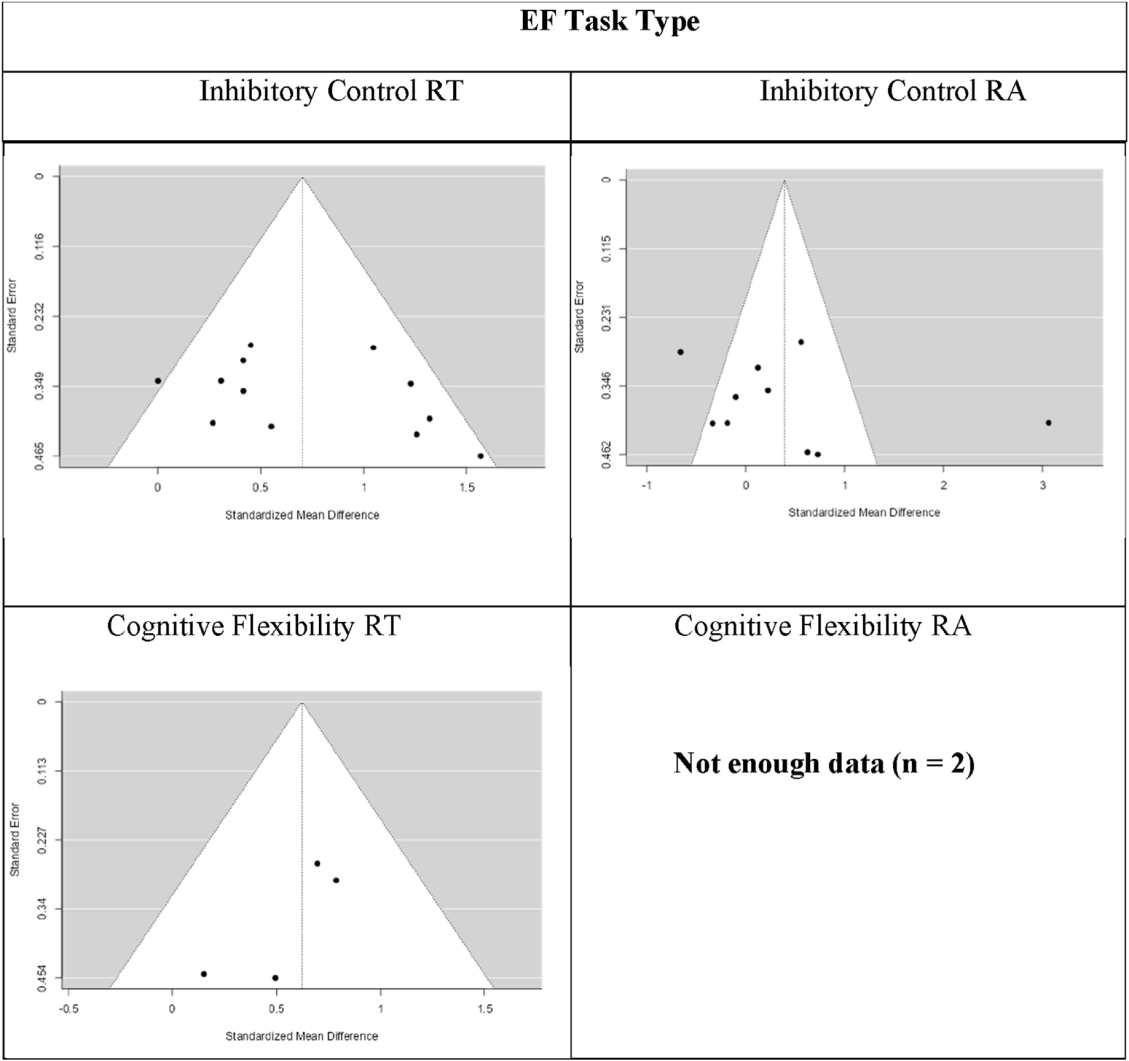

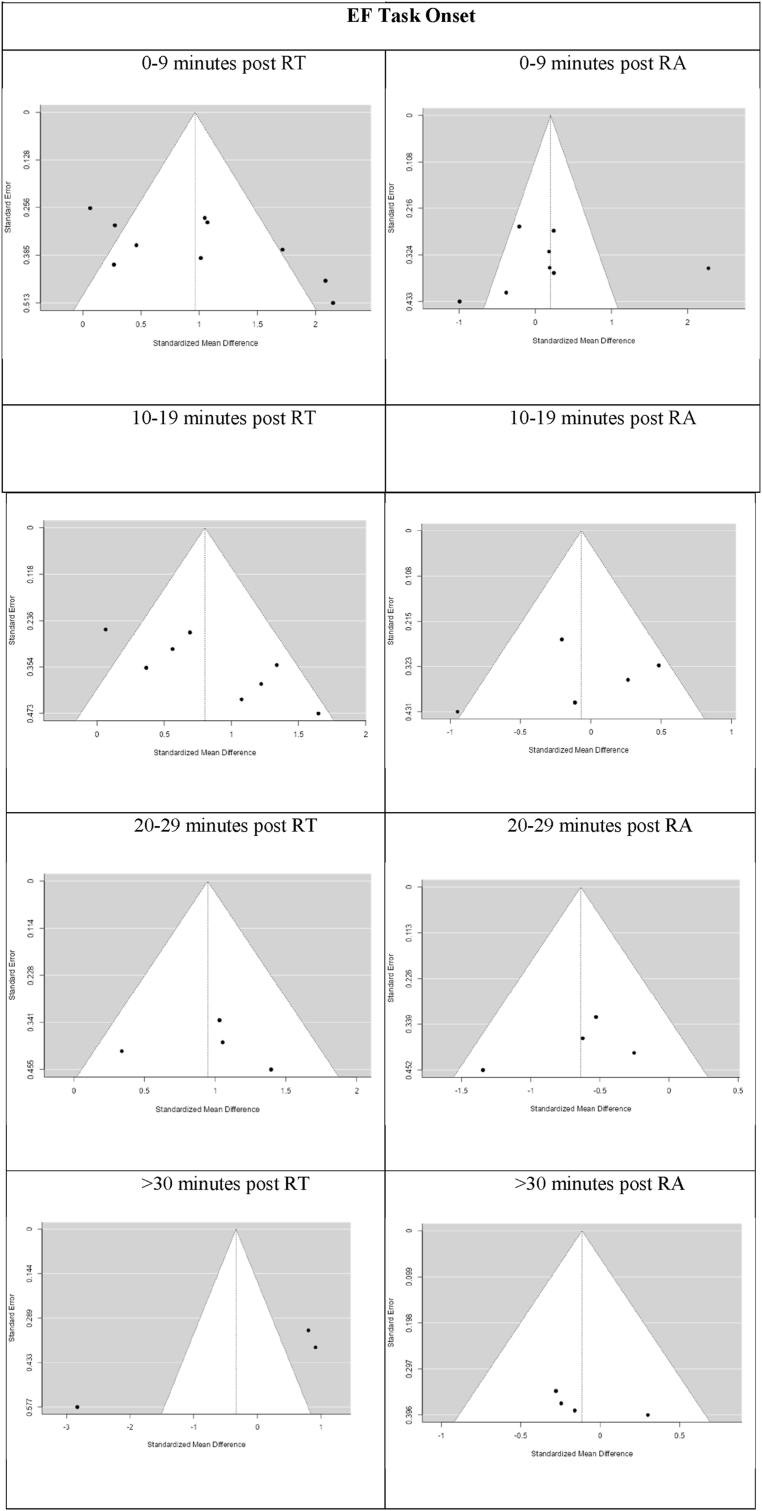


**Figure:**
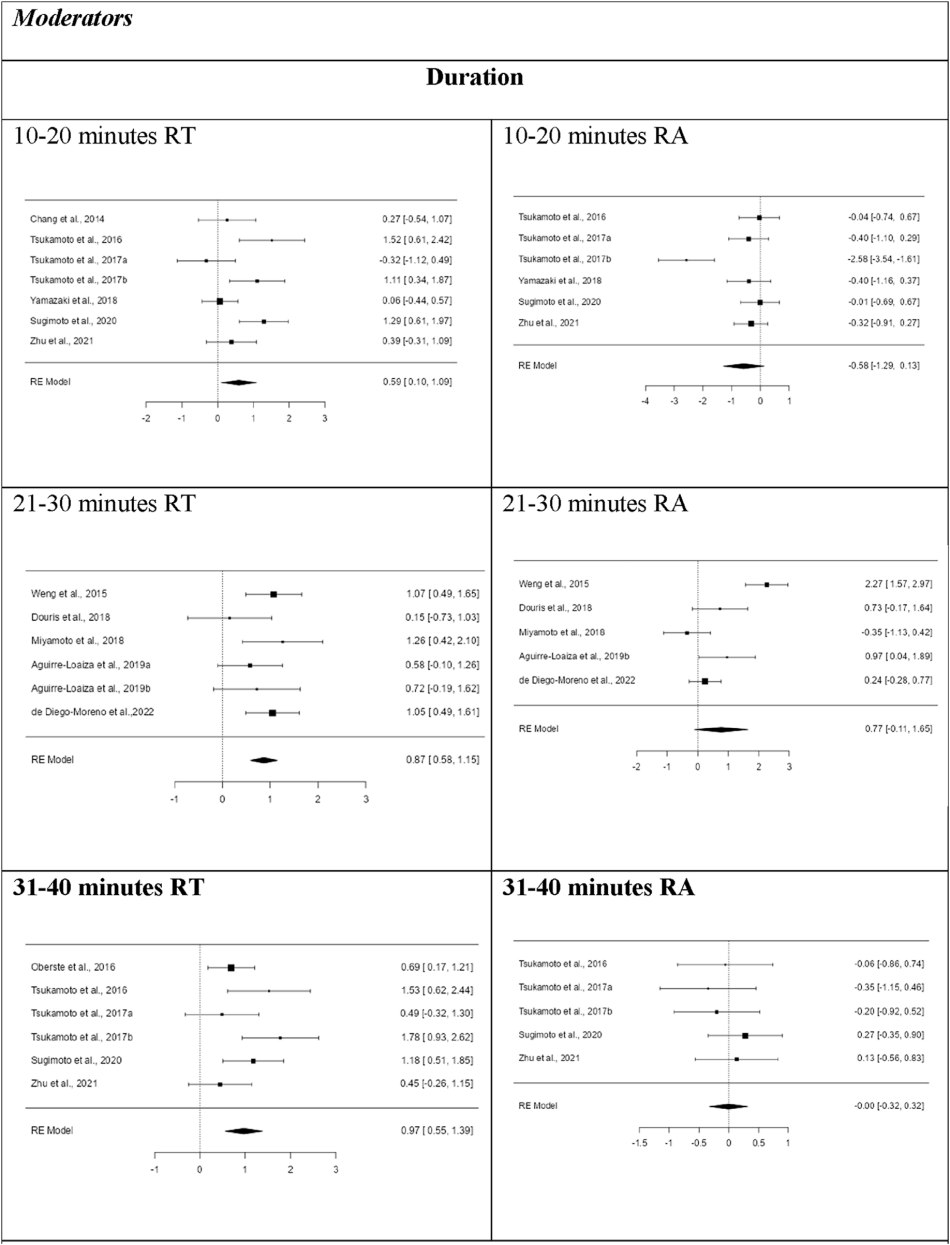

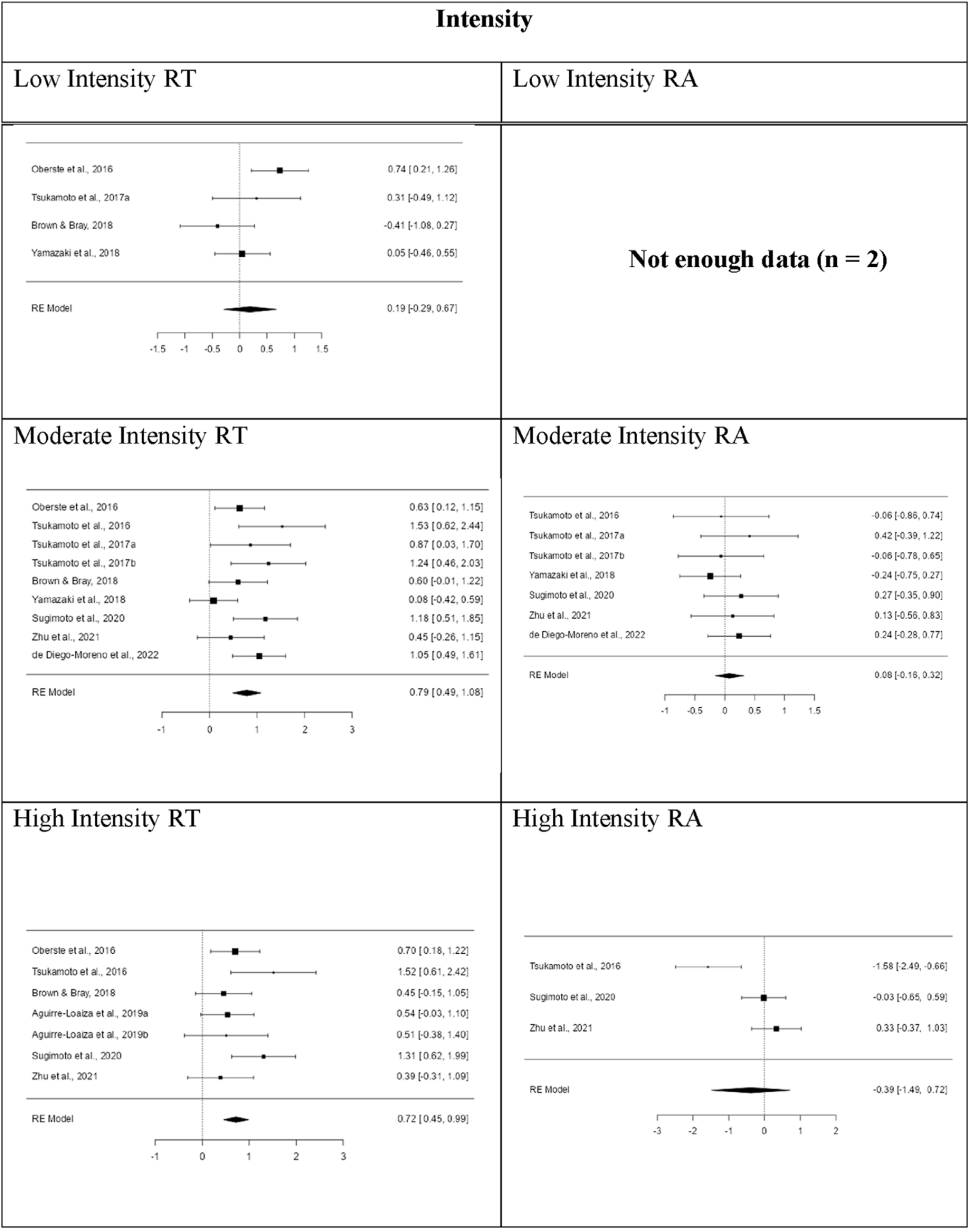

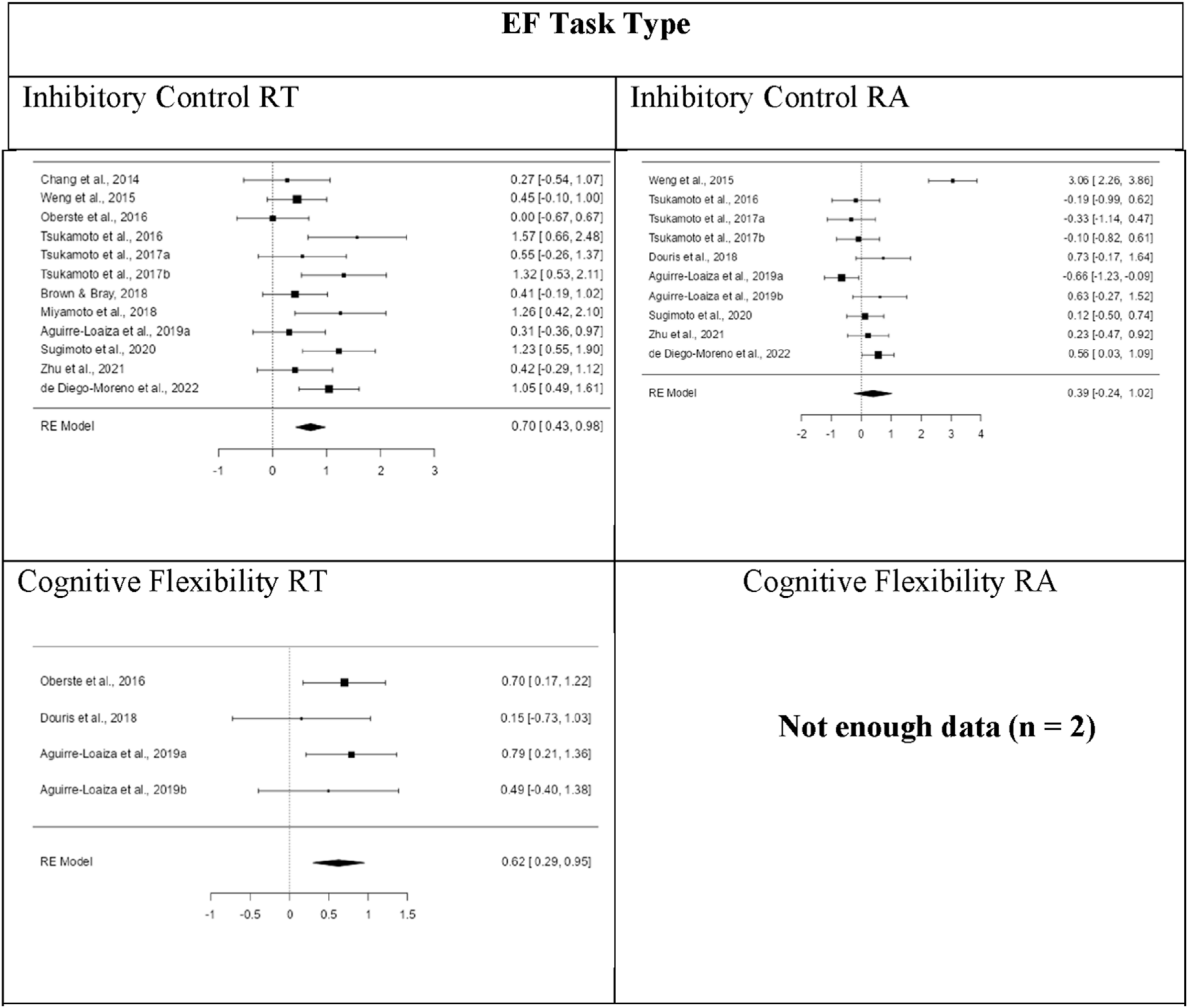

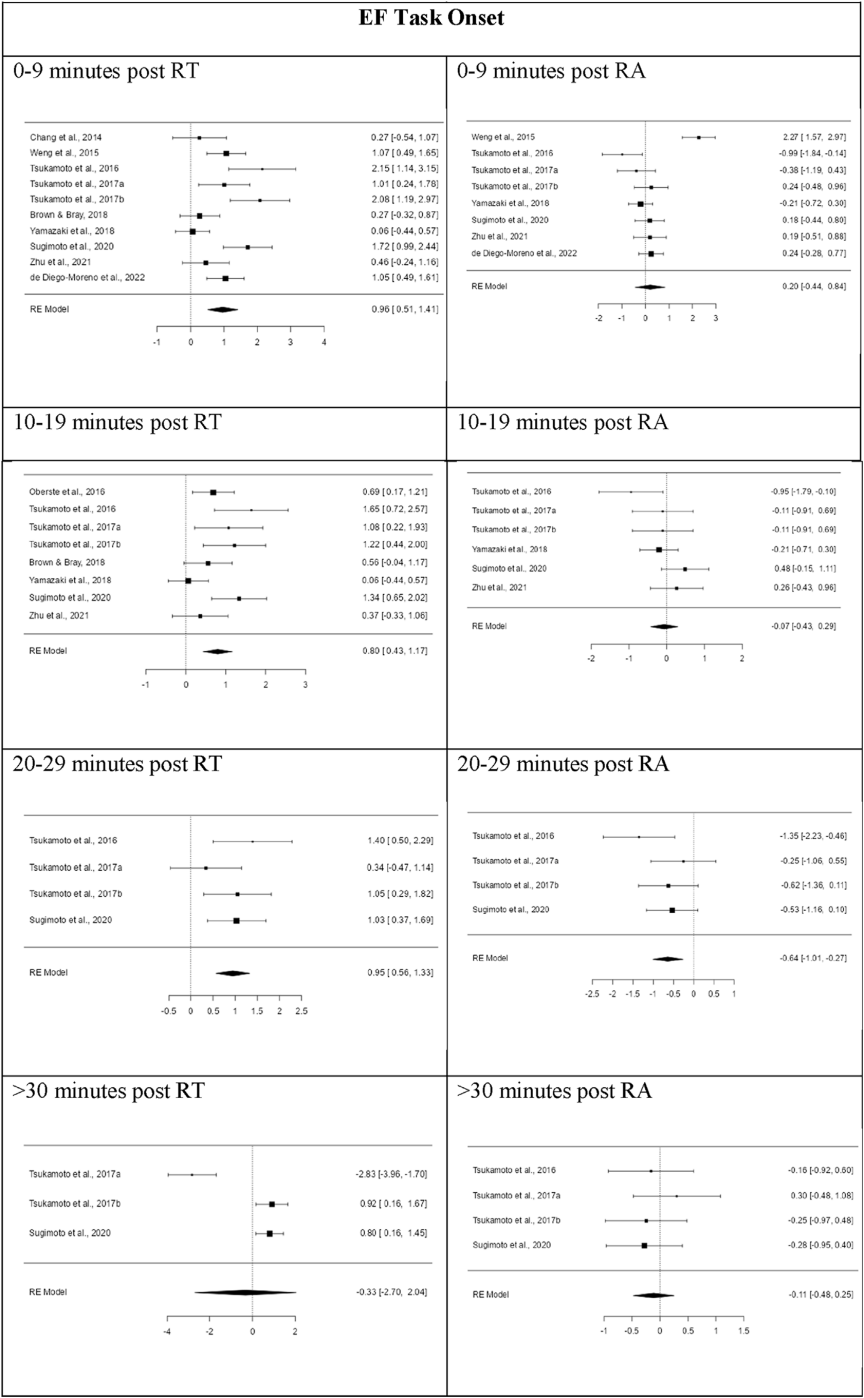


## References

[1] Chang Y.K., Labban J.D., Gapin J.I. (2012). The effects of acute exercise on cognitive performance: a meta-analysis. Brain Res.

[2] Basso J.C., Suzuki W.A. (2017). The effects of acute exercise on mood, cognition, neurophysiology, and neurochemical pathways: a review. Brain Plast.

[3] Moreau D., Chou E. (2019). The acute effect of high intensity exercise on executive function: a meta-analysis. Perspect Psychol Sci.

[4] Miyake A., Friedman N.P., Emerson M.J. (2000). The unity and diversity of executive functions and their contributions to complex "Frontal Lobe" tasks: a latent variable analysis. Cogn psychol.

[5] Huston, Steiner (2016). https://www.elsevier.com/books-and-journals/book-series/handbook-of-behavioral-neuroscience.

[6] Smith E.E., Jonides J. (1997). Working memory: a view from neuroimaging. Cognit Psychol.

[7] Weiner B. (2000). Intrapersonal and interpersonal theories of motivation from an attributional perspective. Educ Psychol Rev.

[8] Basso J., Shang A., Elman M. (2015). Acute exercise improves prefrontal cortex but not hippocampal function in healthy adults. J Int Neuropsychol Soc.

[9] Barisic A., Leatherdale S.T., Kreiger N. (2011 May-Jun). Importance of frequency, intensity, time and type (FITT) in physical activity assessment for epidemiological research. Can J Public Health.

[10] American College of Sports Medicine (2021).

[11] Yerkes R.M., Dodson J.D. (1908). The relation of strength of stimulus to rapidity of habit-formation. J Comp Neurol.

[12] Cooper C.J. (1973). Anatomical and physiological mechanisms of arousal, with special reference to the effects of exercise. Ergonomics.

[13] Davey C.P. (1973). Physical exertion and mental performance. Ergonomics.

[14] McMorris T., Hale B.J. (2012). Differential effects of differing intensities of acute exercise on speed and accuracy of cognition: a meta-analytical investigation. Brain Cognit.

[15] McMorris T., Barwood M., Corbett J. (2018). Central fatigue theory and endurance exercise: toward an interoceptive model. Neurosci Biobehav Rev.

[16] Craig A.D. (2002). How do you feel? Interoception: the sense of the physiological condition of the body. Nat Rev Neurosci.

[17] McMorris T. (2021). The acute exercise-cognition interaction: from the catecholamines hypothesis to an interoception model. Int J Psychophysiol.

[18] Bogdanis G.C., Mallios V.J., Katsikas C. (2021). Effects of exercise structure and modality on physiological and perceptual responses to exercise. J Strength Con Res.

[19] Lambourne K., Tomporowski P. (2010). The effect of exercise-induced arousal on cognitive task performance: a meta-regression analysis. Brain Res.

[20] Kunzler M.R., Carpes F.P. (2022). Moderate intensity cycling combined with cognitive dual-task improves selective attention. Int J Sports Med.

[21] Aguirre-Loaiza H., Arenas J., Arias I. (2019). Effect of acute physical exercise on executive functions and emotional recognition: analysis of moderate to high intensity in young adults. Front Psychol.

[22] Tsukamoto H., Takenaka S., Suga T. (2017). Impact of exercise intensity and duration on postexercise executive function. Med Sci Sports Exerc.

[23] Zhu Y., Sun F., Chiu M.M. (2021). Effects of high intensity interval exercise and moderate intensity continuous exercise on executive function of healthy young males. Physiol Behav.

[24] Tsukamoto H., Suga T., Takenaka S. (2016). Greater impact of acute high intensity interval exercise on post-exercise executive function compared to moderate intensity continuous exercise. Physiol Behav.

[25] Byun K.H., Hyodo K., Suwabe K. (2014). Positive effect of acute mild exercise on executive function via arousal-related prefrontal activations: an fNIRS study. Neuroimage.

[26] Anderson-Hanley C., Arciero P.J., Brickman A.M. (2012). Exergaming and older adult cognition: a cluster randomized clinical trial. Am J Prev Med.

[27] Knaepen K., Goekint M., Heyman E.M. (2010). Neuroplasticity - exercise-induced response of peripheral brain-derived neurotrophic factor: a systematic review of experimental studies in human subjects. Sports Med.

[28] Law M., Stewart D., Pollock N. (1998). https://www.canchild.ca/en/canchildresources/resources/quantguide.pdf.

[29] (2021). The Jamovi Project. *Jamovi*..

[30] Sterne J.A., Sutton A.J., Ioannidis J.P. (2011). Recommendations for examining and interpreting funnel plot asymmetry in meta-analyses of randomised controlled trials. BMJ.

[31] (2020). Review Manager Web (RevMan Web).

[32] Higgins J.P.T., Thomas J., Chandler J. (2022). Cochrane Handbook for Systematic Reviews of Interventions.

[33] Cohen J. (1988).

[34] R Core Team (2021). https://cran.r-project.org.

[35] Vietchtbauer W. (2010). Conducting a meta-analyses in R with metaphor package. J Stat Software.

[36] Cochran W.G. (1954). The combination of estimates from different experiments. Biometrics.

[37] Reitan R.M. (1956).

[38] Kirchner W.K. (1958). Age differences in short-term retention of rapidly changing information. J Exp Psychol Gen.

[39] Stroop J. (1935). Studies of interference in serial verbal reactions. J Exp Psychol Gen.

[40] Eriksen B.A., Eriksen C.W. (1974). Effects of noise letters upon identification of a target letter in a non-search task. Percept Psychophys.

[41] Berg E.A. (1948). A simple objective technique for measuring flexibility in thinking. J Exp Psychol Gen.

[42] Brown D.M.Y., Bray S.R. (2018). Acute effects of continuous and high-intensity interval exercise on executive function. J Appl Biobehav Res.

[43] Chang Y.K., Chi L., Etnier J.L. (2014). Effect of acute aerobic exercise on cognitive performance: role of cardiovascular fitness. Psychol Sport Exerc.

[44] de Diego-Moreno M., Álvarez-Salvago F., Martínez-Amat A. (2022). Acute effects of high-intensity functional training and moderate-intensity continuous training on cognitive functions in young adults. Int J Environ Res Publ Health.

[45] Douris P.C., Handrakis J.P., Apergis D. (2018). The effects of aerobic exercise and gaming on cognitive performance. J Hum Kinet.

[46] Hashimoto T., Tsukamoto H., Takenaka S. (2018). Maintained exercise-enhanced brain executive function related to cerebral lactate metabolism in men. Faseb J.

[47] Miyamoto T., Hashimoto S., Yanamoto H. (2018). Response of brain-derived neurotrophic factor to combining cognitive and physical exercise. Eur J Sport Sci.

[48] Oberste M., Bloch W., Hübner S.T., Zimmer P. (2016). Do reported effects of acute aerobic exercise on subsequent higher cognitive performances remain if tested against an instructed self-myofascial release training control group? A randomized controlled trial. PLoS One.

[49] Sugimoto T., Suga T., Tsukamoto H. (2020). Similar improvements in inhibitory control following low-volume high intensity interval exercise and moderate intensity continuous exercise. Psychol Sport Exerc.

[50] Wang C.C., Shih C.H., Pesce C. (2015). Failure to identify an acute exercise effect on executive function assessed by the Wisconsin card sorting test. Sport Health Sci.

[51] Weng T.B., Pierce G.L., Darling W.G. (2015). Differential effects of acute exercise on distinct aspects of executive function. Med Sci Sports Exerc.

[52] Yamazaki Y., Sato D., Yamashiro K. (2018). Inter-individual differences in working memory improvement after acute mild and moderate aerobic exercise. PLoS One.

[53] McMorris T., Sproule J., Turner A., Hale B.J. (2011 Mar 1). Acute, intermediate intensity exercise, and speed and accuracy in working memory tasks: a meta-analytical comparison of effects. Physiol Behav.

[54] McMorris T. (2020 Oct). Cognitive fatigue effects on physical performance: the role of interoception. Sports Med.

[55] McMorris T. (2009). Exercise and cognitive function. A Neuroendocrinological Explanation.

[56] Miller E.K., Cohen J.D. (2001). An integrative theory of prefrontal cortex function. Annu Rev Neurosci.

[57] Diamond A. (2013). Executive functions. Annu Rev Psychol.

[58] Howie E.K., Pate R.R. (2012). Physical activity and academic achievement in children: a historical perspective. J Sport Health Sci.

[59] Martins R.M.G., Duncan M.J., Clark C.C.T. (2021). The acute effects of continuous and intermittent cycling on executive function in children. Acta Psychol.

[60] Bérdi M., Köteles F., Szabo A. (2011). Placebo effects in sport and exercise: a meta-analysis. Eur J Ment Health.

